# Enhanced liver but not muscle OXPHOS in diabetes and reduced glucose output by complex I inhibition

**DOI:** 10.1111/jcmm.15238

**Published:** 2020-04-06

**Authors:** Miriayi Alimujiang, Xue‐ying Yu, Mu‐yu Yu, Wo‐lin Hou, Zhong‐hong Yan, Ying Yang, Yu‐qian Bao, Jun Yin

**Affiliations:** ^1^ Department of Endocrinology and Metabolism Shanghai Clinical Center for Metabolic Diseases Shanghai Key Laboratory of Diabetes Mellitus Shanghai Diabetes Institute Shanghai Jiao Tong University Affiliated Sixth People’s Hospital Shanghai China; ^2^ Department of Chemistry Shanghai Jiao Tong University School of Medicine Shanghai China; ^3^ Department of Endocrinology and Metabolism Shanghai Eighth People's Hospital Shanghai China

**Keywords:** diabetes, electron transport chain, insulin resistance, liver steatosis, NAFLD, obesity, ROS

## Abstract

Mitochondrial function is critical in energy metabolism. To fully capture how the mitochondrial function changes in metabolic disorders, we investigated mitochondrial function in liver and muscle of animal models mimicking different types and stages of diabetes. Type 1 diabetic mice were induced by streptozotocin (STZ) injection. The db/db mice were used as type 2 diabetic model. High‐fat diet‐induced obese mice represented pre‐diabetic stage of type 2 diabetes. Oxidative phosphorylation (OXPHOS) of isolated mitochondria was measured with Clark‐type oxygen electrode. Both in early and late stages of type 1 diabetes, liver mitochondrial OXPHOS increased markedly with complex IV‐dependent OXPHOS being the most prominent. However, ATP, ADP and AMP contents in the tissue did not change. In pre‐diabetes and early stage of type 2 diabetes, liver mitochondrial complex I and II‐dependent OXPHOS increased greatly then declined to almost normal at late stage of type 2 diabetes, among which alteration of complex I‐dependent OXPHOS was the most significant. In contrast, muscle mitochondrial OXPHOS in HFD, early‐stage type 1 and 2 diabetic mice, did not change. In vitro, among inhibitors to each complex, only complex I inhibitor rotenone decreased glucose output in primary hepatocytes without cytotoxicity both in the absence and presence of oleic acid (OA). Rotenone affected cellular energy state and had no effects on cellular and mitochondrial reactive oxygen species production. Taken together, the mitochondrial OXPHOS of liver but not muscle increased in obesity and diabetes, and only complex I inhibition may ameliorate hyperglycaemia via lowering hepatic glucose production.

## 1 INTRODUCTION

Mitochondria are the main cellular sites devoted to energy metabolism, where most energy‐rich compound ATP is synthesized. Thus, mitochondria are regarded as the powerhouse of cells. Due to its role in energy metabolism, mitochondrial dysfunction is linked with development of metabolic disorders, such as diabetes and obesity. However, current understanding of mitochondrial function in diabetes is controversial. Several human studies revealed that activity of mitochondrial respiratory chain reduced in muscle biopsies from type 2 diabetic patients.^1^, ^2^, ^3^ Following reports indicated that mitochondrial dysfunction may be a consequence rather than cause of insulin resistance and type 2 diabetes.^4^, ^5^, ^6^ In contrast, some studies showed that patients or rats with type 2 diabetes had normal muscle mitochondrial function.^7^, ^8^, ^9^ Muscle mitochondrial function is also widely investigated in high‐fat diet (HFD)‐induced obese and pre‐diabetic conditions, and the findings are also contradictory, as mitochondrial function has been reported unchanged,^10^ increased^11^, ^12^ or deceased.^13^, ^14^ Additionally, it is noteworthy that data on muscle mitochondrial function/dysfunction in type 1 diabetes are sparse.

Compared to numerous studies focusing on the activity of mitochondrial respiration in skeletal muscle, reports about liver mitochondrial function in diabetes were limited and the findings were also inconsistent, with various results indicating the function decreased,^15^ unchanged^16^ or increased.^17^, ^18^ As different studies used different experimental paradigms and approaches to address the issue, it is difficult to integrate all the inconsistent findings on this subject.

The aim of this study was to get a clear picture of mitochondrial functional alteration in diabetes mellitus. Therefore, the disease models in our study include pre‐diabetic obese mice induced with HFD, early stage and late stage of both type 1 and type 2 diabetic mice. The oxidative phosphorylation (OXPHOS) of both liver and skeletal muscle, two of the most important organs in whole energy metabolism, was examined.

Moreover, several lines of evidence proved that insulin sensitizers, including metformin, berberine and thiazolidinediones, were able to exert their anti‐diabetic effects through inhibition of mitochondrial respiratory chain at complex I.^19^, ^20^, ^21^ However, whether inhibition of other complexes than complex I in respiratory chain had beneficial effects on glucose metabolism remained unknown. As our results showed that mitochondrial OXPHOS of liver but not skeletal muscle altered dramatically in the pre‐diabetic and diabetic mice, inhibitors to each complex were tested in the hepatocytes with or without oleic acid (OA) pre‐treatment. Our results indicated only complex I inhibitor rotenone decreased glucose output in the primary hepatocytes without cytotoxicity through changing cellular energy state.

## 2 MATERIALS AND METHODS

### 2.1 Animals

All mice were obtained from SLACCAS (Shanghai, China) and were maintained in a pathogen‐free environment and housed in cages in groups of four to five mice per cage with constant temperature and humidity and 12‐hour light/dark cycle. All animals had free access to water and food at all times except for indicated fasting conditions. All procedures involving the care and use of animals were approved by the ethics committee of Shanghai Jiao Tong University Affiliated Sixth People's Hospital, and all the procedures were performed according to Shanghai Jiao Tong University Affiliated Sixth People's Hospital Guidelines for the Care and Use of Laboratory Animals.

### 2.2 Type 1 diabetic mice

Male C57BL/6J mice (8 weeks) were weighed and fasted overnight prior to STZ injection. STZ (150 mg/kg) was administrated to induce type 1 diabetes. Briefly, STZ was dissolved in freshly prepared sodium citrate buffer (pH 4.5), and STZ solution was injected intraperitoneally immediately after preparation. For the early stage of type 1 diabetes, the following experiments were conducted 3 weeks after STZ injection. For the late stage of type 1 diabetes, the following experiments were conducted 3 months after STZ injection.

### 2.3 Type 2 diabetic mice

For early‐stage type 2 diabetic animal model, 12‐week‐old male C57BL/Ks db/db (db/db) mice, genetically obese and diabetic mice with leptin receptor deficiency, were used. For late stage of type 2 diabetic animal model, 42‐week‐old male db/db mice were used. Corresponding control mice were male C57BL/6J mice with same age.

### 2.4 Pre‐diabetic obese mice

C57BL/6J male mice of 7 weeks old were randomized and fed with HFD (59% of its calories derived from fat, 15% from protein and 26% from carbohydrate, TP24220, Trophic Diet, China) or regular chow diet (CD, 1010041, Jiangsu Xietong Pharmaceutical Bioengineering Co., Ltd.) for 6 months. Bodyweight and random blood glucose were monitored once a week or every 2 weeks.

### 2.5 The intraperitoneal glucose tolerance test (IPGTT) and insulin tolerance test (ITT)

Mice were fasted overnight (12 hours) before glucose injection (1 g/kg) for IPGTT and were fasted for 6 hours prior to insulin injection (1 U/kg for type 1 diabetic mice, and 1.5 U/kg for type 2 diabetic and pre‐diabetic obese mice) for ITT. Blood glucose levels were measured using tail vein blood with Roche glucometer at 0, 15, 30, 60 and 120 minutes after the injection.

### 2.6 Isolation of liver and muscle mitochondria

Animals were killed by cervical dislocation, and liver and skeletal muscle of hind legs (quadriceps femoris, biceps femoris, soleus, gastrocnemius) were removed rapidly. Intact mitochondria were isolated by differential centrifugation, as previously described.^22^, ^23^ Mitochondrial protein concentration was measured using the Bradford Protein Assay Kit (Beyotime) following the manufacturer's instruction.

### 2.7 Mitochondrial oxygen consumption

Mitochondrial oxygen consumption rate (OCR) was measured at 37°C using a Clark‐type oxygen electrode (Strathkelvin 782 Oxygen System) as previously described.^24^ Briefly, all measurements were performed in 0.5 mL oxygen electrode buffer (100 mmol/L KCl, 50 mmol/L Mops, 1.0 mmol/L EGTA, 5.0 mmol/L Kpi, 1 mg/ml defatted BSA, pH 7.4 for liver mitochondria; 10 mmol/L Tris/HCl, 5 mmol/L MgCl_2_, 5.0 mmol/L Kpi, 0.02 mmol/L EGTA/Tris, 0.25 mol/L sucrose, pH 7.4 for muscle mitochondria). Complex I‐dependent OCR was measured in the presence of its substrates glutamate (20 mmol/L) and malate (5 mmol/L), complex II‐dependent OCR was measured in the presence of succinate (20 mmol/L) with rotenone (7.5 μmol/L), and complex IV‐dependent OCR was measured in the presence of TMPD/Asc (1 mmol/L/10 mmol/L) with rotenone (7.5 μmol/L). After mitochondria were added to the chamber, state 2 respiration was started when ADP (final concentration 0.1 mmol/L) was injected. State 3 respiration was initiated by adding referred substrates and 0.2 mmol/L ADP (final concentration). OXPHOS capacity, indicating maximum ADP‐unlimited rate of state 3, was determined by adding 2 mmol/L ADP for evaluation of O_2_ consumption exerted by the maximal ATP synthesis (ATP synthase coupled with a transmembrane proton transport). Electron transport chain (ETC) capacity was measured in the presence of 0.2 mmol/L DNP (uncoupler) to evaluate the uncoupled respiration rate, which was not limited by the capacity of ATP synthesis. OCRs were calculated using strathkelvin 782 system and expressed as nmol/L atoms of O_2_/minute/mg of mitochondrial protein.

### 2.8 Citrate synthase activity

Citrate synthase (CS) activity of liver and muscle mitochondria was measured as described previously ^25^, ^26^ with minor modifications. Mitochondria were treated with 5% cholate (pH 7.0) before adding to the reagent cocktail. CS activity was expressed as nmol/min/mg of mitochondrial protein.

### 2.9 Measurement of adenine nucleotide levels

The early‐stage STZ‐induced type 1 diabetic mice and the corresponding controls were fasted overnight, and the specimens were obtained following anaesthesia with 1% amobarbital intraperitoneally injected. After adequate anaesthesia, the mice were placed supine on an operation table, and a celiotomy was performed to excise liver segments sharply. The liver segment was put into microtube and immersed into liquid nitrogen immediately. For minimizing the degradation of adenine nucleotides (especially ATP), this procedure was completed within 4 seconds. Then, the muscle segment was taken from hind legs. After being frozen in liquid nitrogen for more than 4h, specimens were stored at −80°C. Specimens (200 mg) were homogenized into 1ml of 6% (v/v) ice‐cold HClO_4_ and centrifuged at 10 000 g for 10 minutes at 4°C. The supernatant was neutralized with NaHCO_3_ and filtered prior to determination by high‐performance liquid chromatography (HPLC).

### 2.10 Oil Red O staining

When the mice were killed, liver sections of each mouse were fixed using 4% paraformaldehyde, and then sent to Wuhan Goodbio technology CO., LTD, where they were sectioned and stained with haematoxylin and eosin (H&E) or frozen and stained with oil red O. The slides were observed and photographed under the microscope right after being stained.

### 2.11 Primary hepatocytes isolation

Primary hepatocytes were isolated from male C57BL/6J mice of 8‐10 weeks old by a two‐step perfusion method as previously described with some modifications.^27^, ^28^ Briefly, the liver was perfused with 40‐50 mL of 0.01 mol/L Hepes buffer (pH 7.4) containing 0.05 mmol/L EDTA, followed by 30‐40 mL of a collagenase type‐IV solution (0.5 mg/mL) in 0.025 mol/L Hepes (pH 7.6). The isolated liver cells were centrifuged at 50 g for 10 minutes and washed once with fresh medium. The cells were resuspended with 5 mL DMEM + 0.5 mL 10*PBS + 4.5 mL Percoll and were centrifuged at 70 g for 10 minutes to remove the dead cells. After that, cells were plated on 0.2% gelatin‐coated twelve‐well plates in low glucose DMEM (Gibco) containing 10% FBS and 1% penicillin‐streptomycin mixture. The viability of the primary hepatocytes preparation was at least 85% (as determined by trypan blue exclusion before plating cells). After 4‐6 hours of attachment, the medium was replaced by M199 medium (Gibco) supplemented with antibiotics and 100 nmol/L dexamethasone (Sigma‐Aldrich).

### 2.12 Glucose output assay and Lactate dehydrogenase (LDH) cytotoxicity assay

Primary hepatocytes on 12‐well plates were pre‐treated with M199 medium containing antibiotics and 100 nmol/L dexamethasone for 16h overnight before the glucose output assay. After 16 hours, the cells were rinsed with PBS once and then treated with phenol red‐free, glucose‐free DMEM containing substrates needed for gluconeogenesis (20 mmol/L lactate, 2 mmol/L pyruvate) and inhibitors of each mitochondrial complex at different concentrations indicated in the figure. After the cells were treated for 6 hours, 50 μL culture medium was taken to measure the glucose concentration. LDH contents in the medium and cells were detected simultaneously using the culture medium with a LDH‐Cytotoxicity Assay Kit (Beyotime) as instructed.

### 2.13 Fatty acid treatment

OA was dissolved in H_2_O via being incubated at 75°C thermostatic water bath; then, OA was bound with fatty acid‐free BSA to gain 40 mmol/L OA (with 10% BSA) stock solution. OA was diluted in DMEM, and the cells were incubated in the presence or absence of OA at the concentration of 1 mmol/L (with 0.25% BSA) for 20 hours.

### 2.14 Measurement of ATP/ADP ratio in primary hepatocytes

Primary hepatocytes were cultured and treated as described above. The ATP content and ATP/ADP ratio were measured using bioluminescence assay (Abcam catalog #65313, USA) following manufacturer's instruction.

### 2.15 Detection of cellular and mitochondrial ROS

Prior to 20 μmol/L 2′,7′‐dichlorofluorescin diacetate (DCFDA, Abcam catalog #113851) treatment for 45 minutes, the primary hepatocytes were treated with indicated inhibitors in the medium for glucose output determination. The cellular ROS was measured with excitation at 485 nm (Ex bandwidth: 9 nm) and emission at 535 nm as instructed. The mitochondrial ROS was detected after the hepatocytes were incubated with 2.5 μmol/L MitoSOX Red (Thermo Fisher Scientific, catalog #M36008) for 10 minutes, and fluorescence was detected at 510 nm excitation (Ex bandwidth: 9 nm) and 595 nm emission wavelengths as manufacturer's instruction.

### 2.16 Western blot analysis

After treatment with indicated inhibitors of the complexes, the primary hepatocytes were washed twice with ice‐cold PBS and lysed with RIPA (Beyotime) supplemented with protease and phosphatase inhibitor cocktail (Roche). The lysates were boiled at 95°C for 10 minutes and separated by SDS‐polyacrylamide gel electrophoresis (SDS‐PAGE). Then, the separated proteins were transferred onto a nitrocellulose membrane (GE Healthcare). After blocked with 5% skim milk in the Tris‐Buffered Saline Tween‐20 (TBST) buffer for 1 hour at room temperature, the membrane was probed overnight at 4°C with primary antibody as listed below: phospho‐AMPKα (Thr172) (Cell Signaling Technology 2535), AMPKα (Cell Signaling Technology 5832) and β‐actin (Cell Signaling Technology 3700). Next day, the membrane was washed and re‐blotted with HRP‐conjugated secondary antibody (Cell Signaling Technology) at room temperature for 1h. Chemiluminescent HRP substrate (Millipore) was used to visualize protein bands by electrochemoluminescence (ImageQuant LAS4000). ImageJ was used to quantify the Western signals. Similar procedure was performed on frozen isolated liver mitochondria to detect mitochondrial complex I‐V expression using total OXPHOS rodent WB cocktail (Abcam, ab110413) and VDAC1 (Abcam, ab154856) primary antibody.

### 2.17 Statistical analysis

Data are expressed as means ± SEM. Two‐tailed Student's *t* test and one‐way ANOVA (SPSS 20*.*0, Dunnett's multiple comparisons test for the post hoc test) were used in statistical analysis. A level of *P* < .05 was considered as statistically significant.

## 3 RESULTS

### 3.1 The OCR of liver mitochondria increased at both early stage and late stage of type 1 diabetes

**FIGURE 1:**
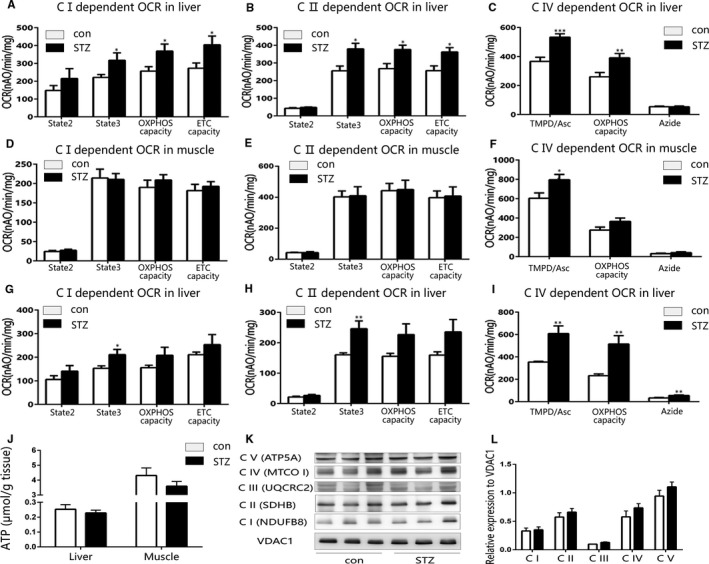
The oxygen consumption rate (OCR) of liver but not muscle mitochondria increased in early‐stage STZ‐induced type 1 diabetic mice. A‐C, The OCR of liver mitochondria isolated from early‐stage type 1 diabetic and control mice. A, Complex I‐dependent OCR. B, Complex II‐dependent OCR. C, Complex IV‐dependent OCR. D‐F, The OCR of muscle mitochondria isolated from early‐stage type 1 diabetic and control mice. D, Complex I‐dependent OCR. E, Complex II‐dependent OCR. F, Complex IV‐dependent OCR. G‐I, The OCR of liver mitochondria isolated from late‐stage type 1 diabetic and control mice. G, Complex I‐dependent OCR. H, Complex II‐dependent OCR. I, Complex IV‐dependent OCR. J, The ATP content of liver and muscle tissue from early‐stage type 1 diabetic and control mice. K, L, Immunoblotting analysis and quantification of protein levels of isolated liver mitochondrial complex components (CI, CII, CIII, CIV, CV) from control and early‐stage STZ‐induced type 1 diabetic mice. Data are expressed as means ± SEM (n = 8). ^*^ *P* < .05, ^**^ *P* < .01, ^***^ *P* < .001 vs control

The quality of isolated mitochondria was evaluated with mitotraker green staining and transmission electron microscopy (TEM). As shown in Figure S1, the isolated liver and muscle mitochondria were stained by mitotraker green, and the TEM images displayed intact mitochondria with ultrastructure. Then, we assessed main functional changes in liver and muscle mitochondria isolated from early‐stage type 1 diabetic mice and found out the liver mitochondrial OCR of type 1 diabetes increased significantly compared with that of control (Figure 1A‐C, each representing complex I‐, II‐ and IV‐dependent respiration. A representative trace of recording complex I‐dependent OCR was shown in Figure S2. However, the skeletal muscle mitochondrial OCR from early‐stage type 1 diabetic mice did not change on all tested substrate conditions, except TMPD/Asc (complex IV substrate)‐stimulating condition (Figure 1D‐F). Besides, the complex II‐dependent calcium retention capacity (CRC) and H_2_O_2_ production of liver mitochondria increased significantly at the early stage of type 1 diabetes (Figure S3F,J), yet complex I‐dependent membrane potential and CS activity in liver mitochondria were not affected (Figure S3H,L), and there were no significant differences of liver lipid accumulation in the early‐stage type 1 diabetic mice (Figure S3N,O). Furthermore, we measured liver mitochondrial function of type 1 diabetic mice at their late stage of disease progress (3 months after STZ injection). The liver mitochondrial complex I‐, II‐dependent state 3 OCR and overall complex IV‐dependent OCR of late‐stage type 1 diabetic mice increased markedly, but the complex I‐ and II‐dependent state 2, OXPHOS capacity and ETC capacity of late‐stage type 1 diabetic mice only had an increasing tendency without statistic differences (Figure 1G‐I). Above all, the increase of complex IV‐dependent OCR was the most significant and steadiest during disease progression of type 1 diabetes. However, no significant differences in ATP, ADP and AMP contents of liver and skeletal muscle were observed between early‐stage type 1 diabetic and control mice, although the abundance of ATP content in muscle was higher than that in liver (Figure 1J and Figure S4A,C,E,G). We further assessed the protein expression of complex I to V in isolated liver mitochondria from early‐stage type 1 diabetic and control mice, and no significant difference was observed between the two groups (Figure 1K,L), indicating the liver mitochondrial OXPHOS function enhanced without elevated expression of mitochondrial complexes. In addition, there was mild liver lipid accumulation in the late‐stage type 1 diabetic mice compared to control mice (Figure S5J,K).

### 3.2 The liver mitochondrial OCR increased at early stage rather than late stage of type 2 diabetes

**FIGURE 2:**
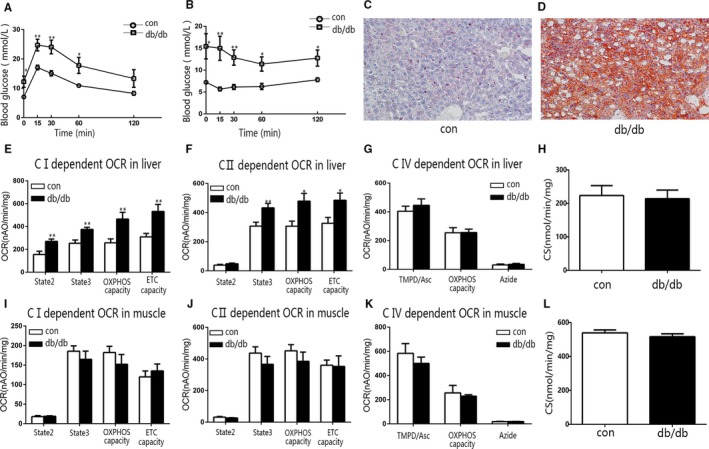
The OCR of liver mitochondria increased in early‐stage db/db mice. A, B, The IPGTT and ITT for early‐stage db/db and control (con) mice. C, D, Representative images showing oil red O staining of liver sections from control (C) and early‐stage db/db (D) mice. E‐G, The OCR of liver mitochondria from early‐stage db/db and control mice. E, Complex I‐dependent OCR. F, Complex II‐dependent OCR. G, Complex IV‐dependent OCR. H, The CS activity of liver mitochondria isolated from early‐stage db/db and control mice. (I‐K) The OCR of muscle mitochondria from early‐stage db/db and control mice. I, Complex I‐dependent OCR. J, Complex II‐dependent OCR. K, Complex IV‐dependent OCR. L, The CS activity of muscle mitochondria isolated from early‐stage db/db and control mice. Data are expressed as means ± SEM (n = 8). ^*^ *P* < .05, ^**^ *P* < .01, ^***^ *P* < .001 vs control

**FIGURE 3:**
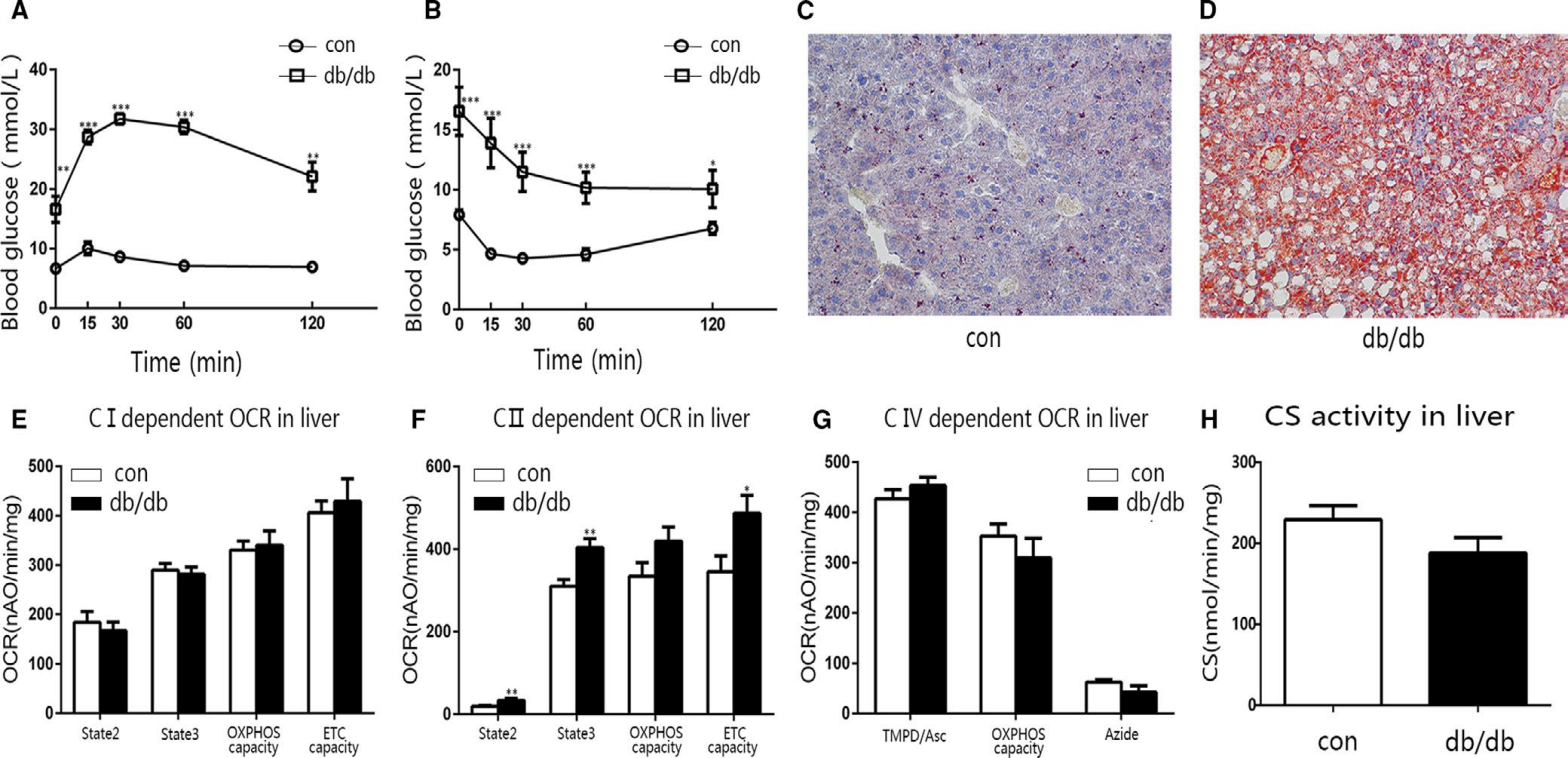
The main characteristics of late‐stage db/db mice. A, B, The IPGTT and ITT for late‐stage db/db and control (con) mice. C, D, Representative images showing oil red O staining of liver sections from control (C) and late‐stage db/db (D) mice. E‐G, The OCR of liver mitochondria from late‐stage db/db and control mice. E, Complex I‐dependent OCR. F, Complex II‐dependent OCR. G, Complex IV‐dependent OCR. H, The CS activity of liver mitochondria isolated from late‐stage db/db and control mice. Data are expressed as means ± SEM (n = 4‐8). ^*^ *P* < .05, ^**^ *P* < .01, ^***^ *P* < .001 vs control

To examine the change of mitochondrial function during type 2 diabetes, we conducted the same experiments applied to type 1 diabetic mice on early stage and late stage of type 2 diabetic db/db mice and the corresponding controls. Impaired glucose tolerance and insulin resistance were observed in both early‐ and late‐stage type 2 diabetes (Figures 2, 3). Notably, the oil red O staining of liver from both early‐stage and late‐stage type 2 diabetic db/db mice presented significant lipid accumulation (Figures 2, 3). At the early stage of type 2 diabetes, the liver mitochondrial complex I‐ and II‐dependent OCRs increased significantly (Figure 2E,F). However, the skeletal muscle mitochondrial OCR from the same animals did not alter (Figure 2I‐K). Meanwhile, the CRC, mitochondrial membrane potential and H_2_O_2_ production of liver and muscle mitochondria from early‐stage type 2 diabetic db/db mice did not alter (Figure S6A‐G). At the late stage of type 2 diabetes, the change of liver mitochondrial OCR was not significant as at the early stage, for only the complex II‐dependent OCR remained statistically significant (Figure 3E‐G). In addition, the liver mitochondrial complex II‐dependent CRC of late‐stage type 2 diabetic db/db mice increased, and the complex I‐dependent membrane potential decreased significantly (Figure S7B,C).

### 3.3 The liver mitochondrial OCR increased after long‐term HFD feeding

**FIGURE 4:**
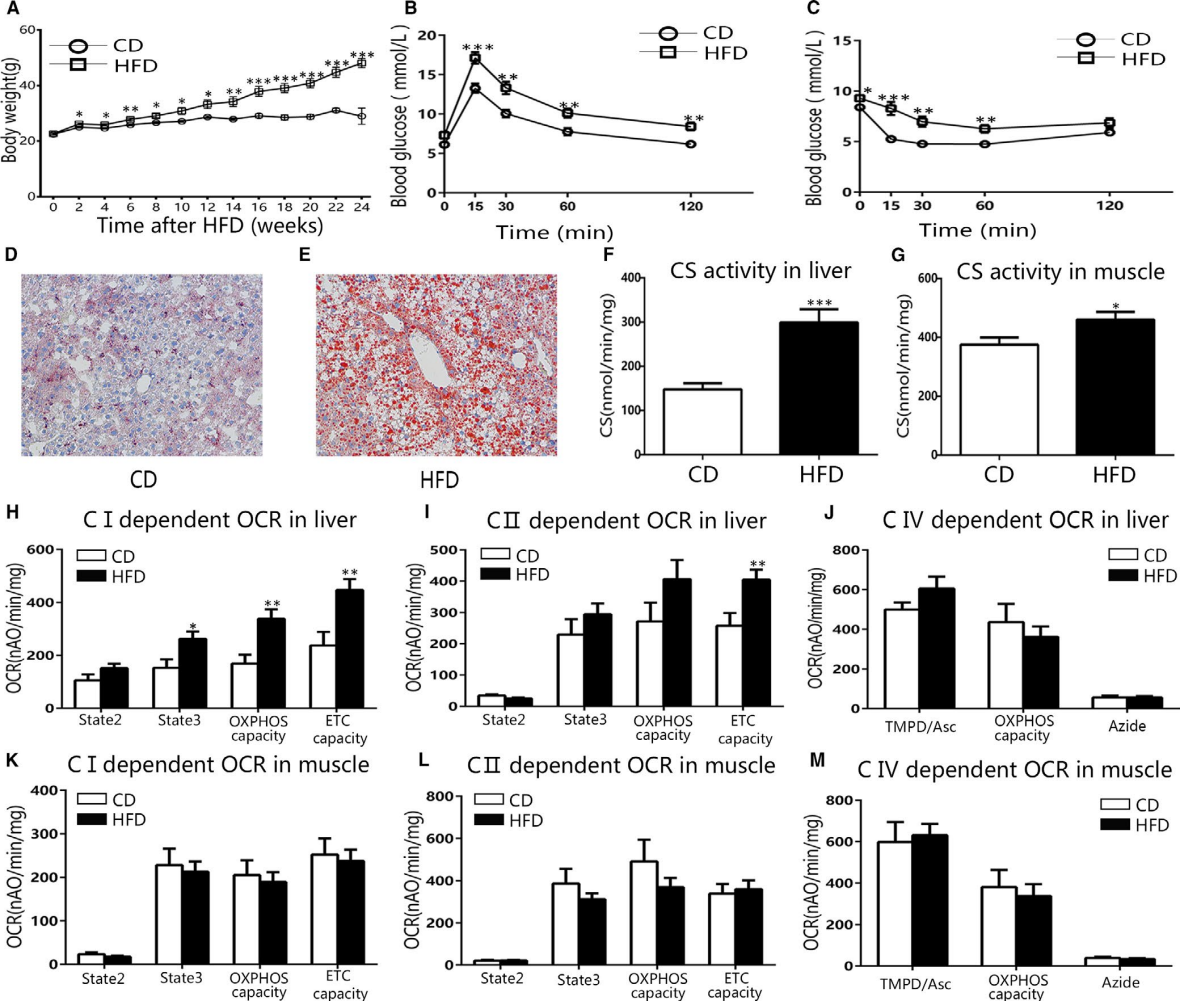
The OCR of liver mitochondria of HFD mice partially increased compared with CD mice. A, The bodyweight of HFD and CD mice. B, C, The IPGTT and ITT for HFD mice and CD controls. D, E, Representative images showing oil red O staining of liver sections from CD (D) and HFD mice (E). F, G, The CS activity of liver and muscle mitochondria isolated from HFD and CD mice. H‐J, The OCR of liver mitochondria from HFD and CD mice. H, Complex I‐dependent OCR. I, Complex II‐dependent OCR. J, Complex IV‐dependent OCR. K‐M, The OCR of muscle mitochondria from HFD and CD mice. K, Complex I‐dependent OCR. L, Complex II‐dependent OCR. M, Complex IV‐dependent OCR. Data are expressed as means ± SEM (n = 10‐15). ^*^ *P* < .05, ^**^ *P* < .01, ^***^ *P* < .001 vs control

We obtained a pre‐diabetic obese model by feeding C57BL/6J male mice with HFD for 6 months. At the end‐point, the average bodyweight of HFD and control mice was 48.1 ± 10.7 vs 29.0 ± 10.5g (*P* < .001, Figure 4A). HFD mice developed insulin resistance and impaired glucose tolerance without elevation of fasting blood glucose levels (Figure 4B,C). Long‐term HFD exposure resulted in significant hepatic lipid accumulation as well (Figure 4D,E), whilst the CS activity in both liver and muscle mitochondria isolated from HFD mice was higher than that from control mice (*P* < .001 and *P* = .0362, Figure 4F,G). In addition, long‐term HFD increased liver complex I‐dependent OCR and complex II‐dependent ETC capacity (Figure 4H,I), whilst had no effects on liver complex IV‐dependent OCR and overall muscle mitochondrial OCR (Figure 4J‐M). The CRC, mitochondrial membrane potential of liver and muscle mitochondria from HFD mice did not changed, but H_2_O_2_ production decreased (Figure S8B‐H).

### 3.4 The mitochondrial complex I inhibitor reduced hepatic glucose output

**FIGURE 5:**
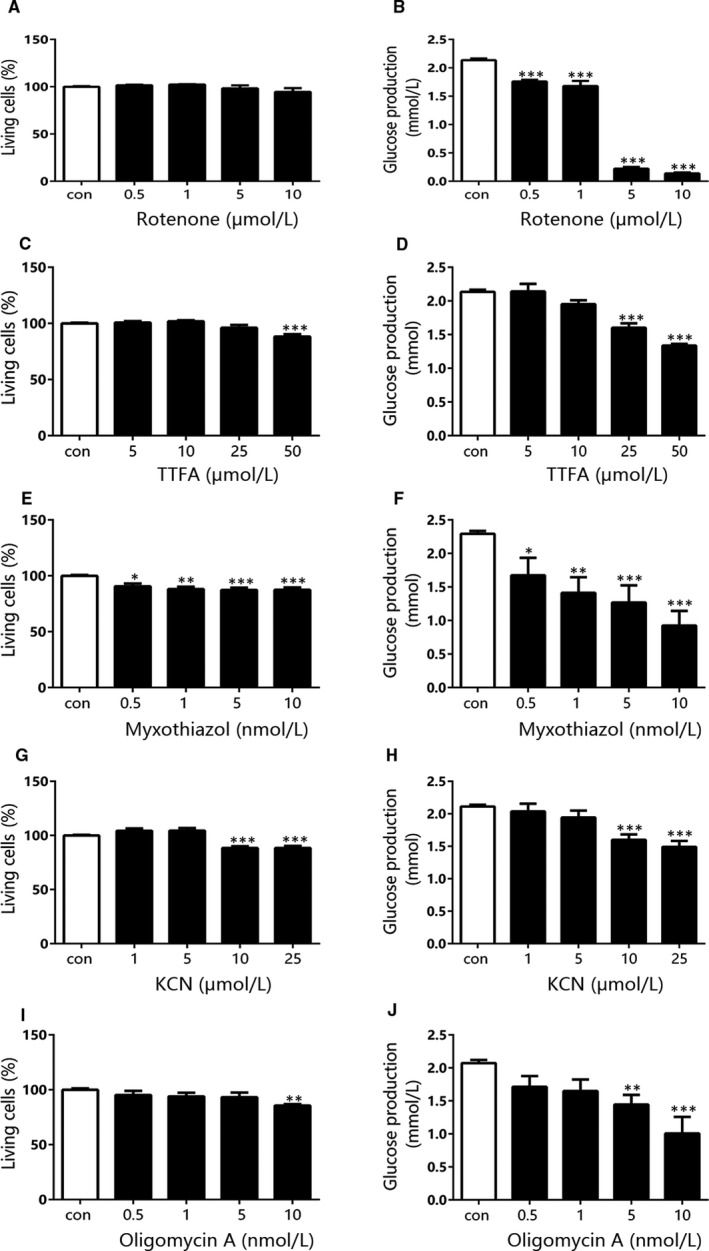
The effect of each complex inhibitor on glucose output and cytotoxicity in mouse primary hepatocytes. The living cell percentage (A) and glucose output (B) of mouse primary hepatocytes after treated with different concentration of rotenone. The living cell percentage (C) and glucose output (D) of mouse primary hepatocytes after treated with different concentration of TTFA. The living cell percentage (E) and glucose output (F) of mouse primary hepatocytes after treated with different concentration of myxothiazol. The living cell percentage (G) and glucose output (H) of mouse primary hepatocytes after treated with different concentration of KCN. The living cell percentage (I) and glucose output (J) of mouse primary hepatocytes after treated with different concentration of oligomycin A. Data are expressed as means ± SEM (n = 4). ^*^ *P* < .05, ^**^ *P* < .01, ^***^ *P* < .001 vs control

**FIGURE 6:**
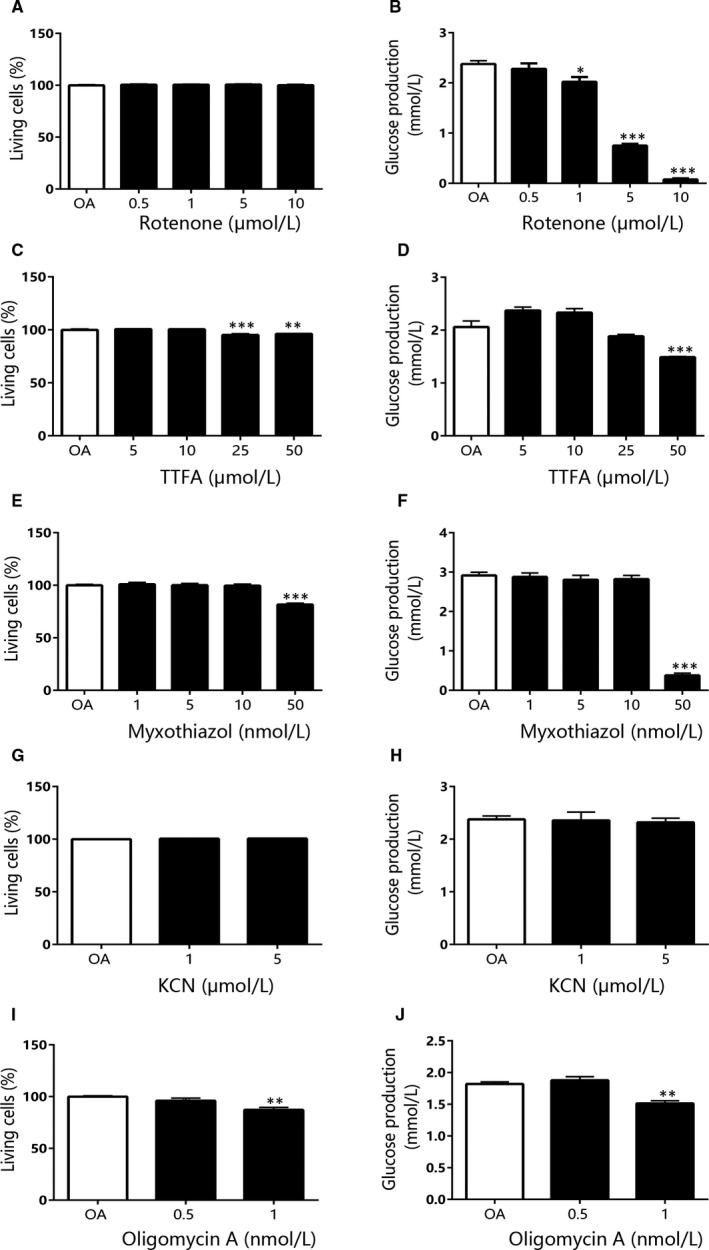
The effect of each complex inhibitor on glucose output and cytotoxicity in mouse primary hepatocytes pre‐treated with 1 mmol/L OA. The living cell percentage (A) and glucose output (B) of mouse primary hepatocytes after treated with different concentration of rotenone. The living cell percentage (C) and glucose output (D) of mouse primary hepatocytes after treated with different concentration of TTFA. The living cell percentage (E) and glucose output (F) of mouse primary hepatocytes after treated with different concentration of myxothiazol. The living cell percentage (G) and glucose output (H) of mouse primary hepatocytes after treated with different concentration of KCN. The living cell percentage (I) and glucose output (J) of mouse primary hepatocytes after treated with different concentration of oligomycin A. Data are expressed as means ± SEM (n = 4). ^*^ *P* < .05, ^**^ *P* < .01, ^***^ *P* < .001 vs control

As enhanced gluconeogenesis was indicated to play an important role in hyperglycaemia of diabetes, and our above findings showed just liver mitochondrial OXPHOS function increased in both type 1 and type 2 diabetes, we explored the effects of inhibitors to each complex in OXPHOS system on hepatic glucose output**.** Meanwhile, the cytotoxicity of each inhibitor in hepatocytes was determined with LDH release. After treatment with rotenone, a specific complex I inhibitor, glucose production in the primary mouse hepatocytes, was strongly suppressed (Figure 5B). In addition, LDH release did not change in the hepatocytes exposed to rotenone for 6 hours with the same dose range (Figure 5A). But the effective dose range was similar to or overlapped the toxic dose of TTFA (complex II inhibitor), myxothiazol (complex III inhibitor), KCN (complex IV inhibitor) and oligomycin A (complex V inhibitor, Figure 5C‐J). We further investigated the effects of the inhibitors on hepatic glucose output in the primary hepatocytes pre‐treated with OA, which developed significant lipid accumulation and enhanced gluconeogenesis (Figure S9A‐C). The findings were consistent with the results in the absence of OA and reconfirmed that only rotenone was able to reduce gluconeogenesis without cell damage (Figure 6A‐J). We also examined the effects of the inhibitors on glucose consumption and lactate release in HepG2 cells. Among the compounds, myxothiazol exhibited a similar strong action on induction of glucose consumption and lactate release as rotenone (Figure S10E,F). The in vitro results indicated that inhibition of liver mitochondrial complex I rather than the other complexes may alleviate the enhanced gluconeogenesis in diabetes.

### 3.5 The mitochondrial complex I inhibitor reduced hepatic glucose output through affecting hepatocyte energy state

**FIGURE 7:**
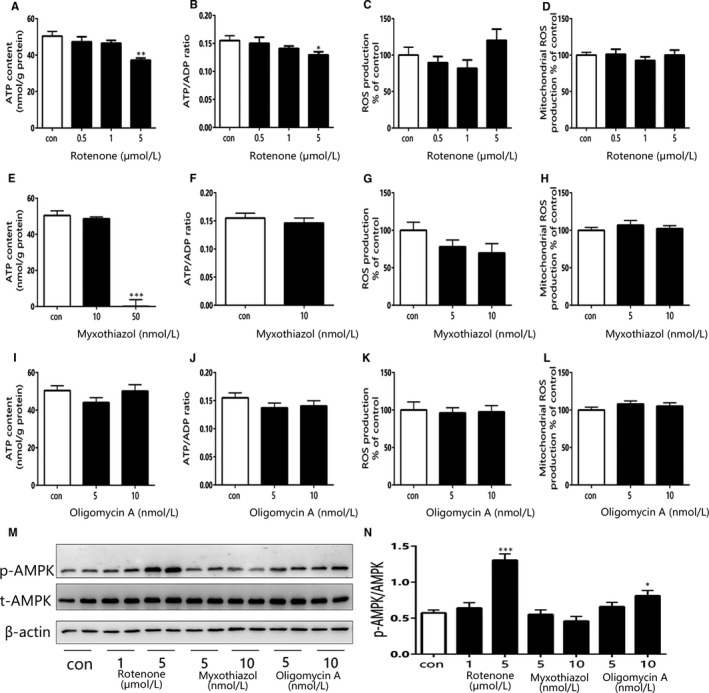
The effects of complex I, III and V inhibitors on cellular energy state and ROS production in mouse primary hepatocytes. A, B, The ATP content and ATP/ADP ratio of primary hepatocytes after treatment with rotenone. C, D, The cellular and mitochondrial ROS production (measured by DCFDA and Mitosox Red, respectively) of primary hepatocytes after treatment with rotenone. E, F, The ATP content and ATP/ADP ratio of primary hepatocytes after treatment with myxothiazol. G, H, The cellular and mitochondrial ROS production of primary hepatocytes after treatment with myxothiazol. H, I, The ATP content and ATP/ADP ratio of primary hepatocytes after treatment with oligomycin A. J, K, The cellular and mitochondrial ROS production of primary hepatocytes after treatment with oligomycin A. Data are expressed as means ± SEM (n = 4‐6). ^*^ *P* < .05, ^**^ *P* < .01, ^***^ *P* < .001 vs control

We further assessed the energetic state, cellular and mitochondrial ROS of primary hepatocytes following treatment with complex I, III and V inhibitors (representative images were shown in Figure S11A,B). The ATP content and ATP/ADP ratio decreased with increasing doses of rotenone and reached a statistically significant difference at 5 μmol/L dosage (Figure 7A,B). Meanwhile, the cellular and mitochondrial ROS production were not affected by same dosages of rotenone (Figure 7C,D). Complex III inhibitor myxothiazol strongly reduced both cellular ATP and ADP contents to nearly zero at 50 nmol/L concentration due to visible cytotoxicity (Figure 7E,F); however, under lower dosage myxothiazol had no effects on both cellular energy state and ROS production (Figure 7G,H). Complex V inhibitor oligomycin A had no effects on ATP content, ATP/ADP ratio, cellular and mitochondrial ROS production at 5 and 10 nmol/L concentrations (Figure 7I‐L). We also evaluated the effects of the inhibitors on AMPK activity, for AMPK has been identified as an indicator of cellular energy status. As shown in Figure 7M,N, 5 μmol/L rotenone resulted in a 2.3‐fold increase of p‐AMPK/AMPK ratio, and 10 nmol/L oligomycin A led to a 1.4‐fold increase of p‐AMPK/AMPK ratio compared with control. These results indicated that the reduced glucose output exerted by inhibiting mitochondrial complex I and V may depend on the reduced cellular energy state rather than ROS production.

## 4 DISCUSSION

One of our significant findings was that the liver mitochondrial OXPHOS function increased to varying degrees in models tested in this study. Previously, liver mitochondrial function in diabetes was seldom investigated, and results were inconsistent. Franko et al^29^ reported that the liver complex I‐dependent OCR increased in STZ‐induced type 1 diabetic mice; however, they did not determine the complex IV‐dependent OCR. Holmström et al^15^ investigated fresh tissue instead of isolated mitochondria and found that the liver mitochondrial respiratory capacity decreased in db/db mice, whereas Buchner et al proved that the amount of mitochondria isolated per gram of liver from obese mice was less than that from lean mice.^30^ Thus, when using same amount of tissue weight to evaluate the respiratory function, the results might be influenced by the different content of mitochondria not by its intrinsic function. In this study, induced OXPHOS was observed in isolated mitochondria from liver in both pre‐diabetic and diabetic mice. We postulate that fuel overload in the liver may account for this alteration. It is known that GLUT2 is the major glucose transporter of hepatocytes in rodents and humans.^31^ Unlike GLUT1, GLUT2 has a high Km value (15‐20 mmol/L), which allows it to transport much more glucose into hepatocytes in the presence of hyperglycaemia.^32^ Furthermore, hyperglycaemia is able to increase GLUT2 mRNA and protein expression in liver.^33^ In addition, high level of free fatty acids in obesity and diabetes provide another type of excess fuel to liver,^34^ which might explain the enhanced mitochondrial OXPHOS function in pre‐diabetic obese mice without hyperglycaemia.

In contrast to liver, we observed that the skeletal muscle mitochondrial OXPHOS function of early‐stage type 1, type 2 and pre‐diabetic mice did not change, which is in accordance with some other studies.^8^, ^10^ We assume that the unchanged fuel load in myocytes might explain this result. It is established that GLUT4 mediates insulin‐stimulated glucose transport in muscle and adipose tissue.^35^ In the development of insulin resistance and type 2 diabetes, GLUT4 fails to translocate to plasma membrane in response to insulin.^36^ Absolute deficiency of insulin in type 1 diabetes or insulin resistance in type 2 diabetes could diminish GLUT4‐mediated uptake of glucose from bloodstream in skeletal muscle, leading to reduced glucose load in myocytes. However, in aforementioned conditions, skeletal muscle shows higher lipid oxidation during post‐absorptive state.^37^ Reduced glucose uptake in the myotubes is compensated by excess triglycerides or free fatty acids in obesity and diabetes. Taken together, it suggests that skeletal muscle is able to keep energy balance even in the severe disturbance of glucose metabolism. Previous studies reported that both muscle‐specific insulin receptor knockout mice and GLUT4‐deficient mice displayed normal glucose tolerance.^38^, ^39^ Holmström et al found that in obese diabetic (db/db) mice, the OXPHOS function of glycolytic muscle increased and that of oxidative muscle decreased.^15^ Since they investigated fresh tissue rather than isolated mitochondrial, and red muscles were rich in mitochondria, myoglobin and oxidative enzymes compared with white muscles,^40^ it was hard to assess the exact mitochondrial function. The isolated mitochondria in this study are from the skeletal muscle of hind legs including glycolytic and oxidative muscle. Thus, the results of our study represent the overall mitochondrial OXPHOS function in the skeletal muscle. Further experiments will address specific features of glycolytic and oxidative fibre mitochondria in the future. In addition, several groups reported that HFD only affected the respiration of muscle mitochondria with fatty acids, and there was no change when substrates like pyruvate/glutamate plus malate and succinate were used.^4^, ^41^, ^42^ In the present study, we did observe that CS activity increased in the muscle mitochondria of HFD‐induced pre‐diabetic mice, indicating the Krebs cycle was enhanced. That suggests HFD may up‐regulate muscle mitochondrial function mildly in the absence of OXPHOS alteration.

The studies on mitochondrial function in diabetes and obesity are numerous, but the findings were always inconsistent due to the different experimental approaches to assess mitochondrial function, different parts of tissue sample or the different types of animal model etc being used, which was systematically reviewed by Pinti et al^43^ recently. The strength of this study is that it covers not only type 1 and 2 diabetes and two major metabolic organs liver and muscle, but also the different stages of diseases. Moreover, all mitochondria isolation and OXPHOS function measurement are conducted in the same system, and the results show only liver mitochondrial function presents dynamic change in pre‐diabetes and diabetes, indicating liver mitochondria are sensitive and flexible to the change of energy homeostasis.

**FIGURE 8:**
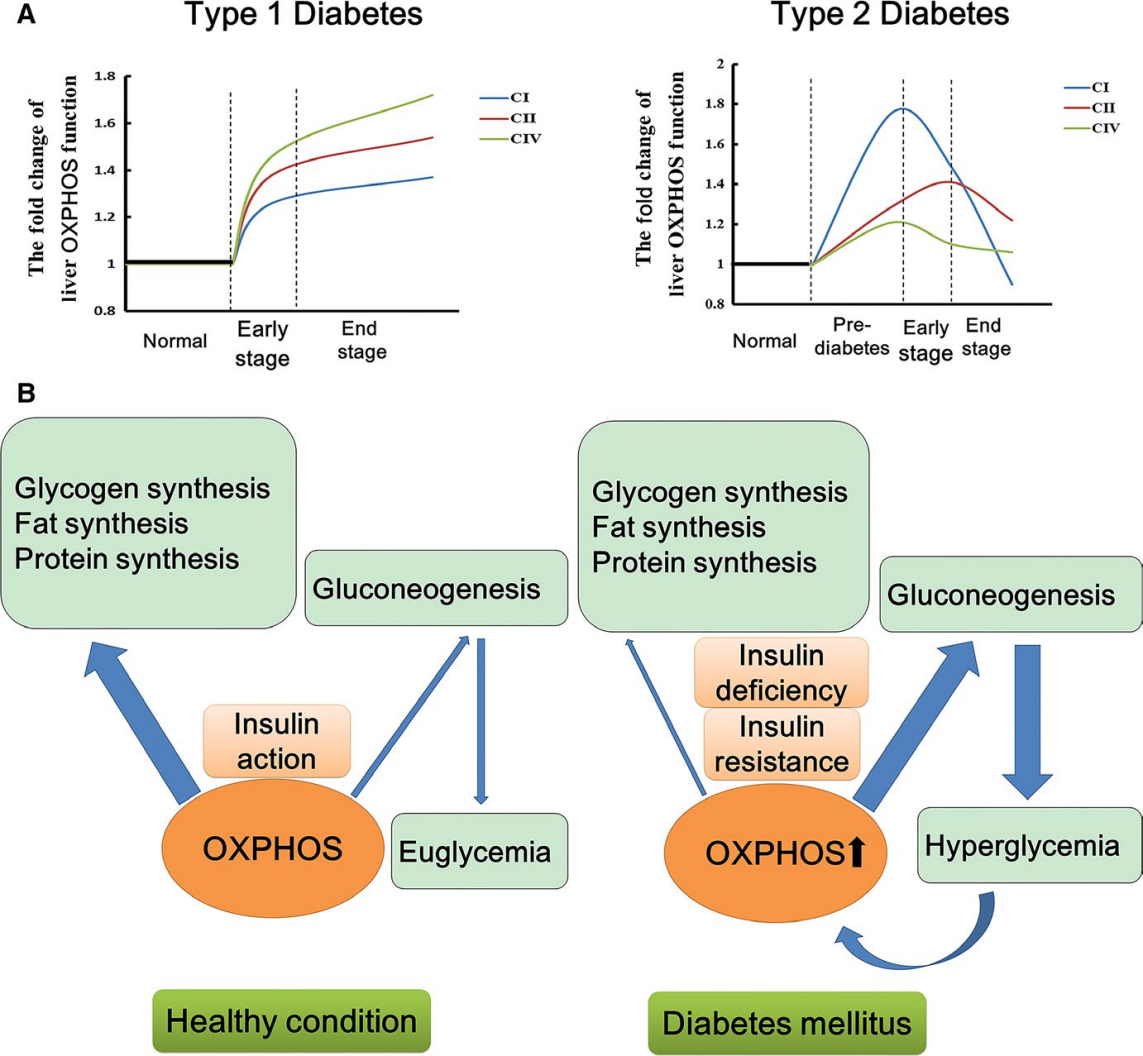
A, The patterns of liver mitochondrial OXPHOS change during type 1 and type 2 diabetes development. The schematic diagram was based on the ratio of liver mitochondrial state 3 respiration of disease models to corresponding controls (shown as Table S1). Briefly, the liver mitochondrial OXPHOS function was increasing continuously during the course of type 1 diabetes, and especially, the increase of complex IV‐dependent OXPHOS function is the most significant. The liver OXPHOS function kept a high level at pre‐diabetes and early stage of type 2 diabetes. Then it declined slowly and returned to nearly normal level at the late stage of type 2 diabetes, among which the change of complex I‐dependent OXPHOS function was the most dramatic, and the complex IV‐dependent OXPHOS function was the least affected. B, The liver OXPHOS contributes differently to anabolism and gluconeogenesis pathways under different energy homeostasis. In healthy condition, majority of ATP produced by OXPHOS fuels anabolism pathway rather than gluconeogenesis (presented by the thick and thin arrows). However, in diabetic condition, liver OXPHOS directs much more ATP to gluconeogenesis than anabolic pathways like glycogen, triglyceride and protein synthesis. Thus, induced liver OXPOHS plays a pivotal role in the vicious circle that hyperglycaemia and gluconeogenesis interacts as both cause and effect

The ATP content in both liver and muscle did not change in early‐stage type 1 diabetic mice. It is known that the dephosphorylation of ATP and rephosphorylation of ADP and AMP occur repeatedly in the course of aerobic metabolism. Our data may suggest a tight balance between ATP synthesis and expenditure in diabetic liver. More ATP produced by overheated OXPHOS leads to more ATP consumption. It is known that hepatic gluconeogenesis, an ATP consuming pathway, dramatically enhances in diabetes.^36^ This study suggests that, in metabolic disorders, glucose and fatty acids rush into liver to over‐activate mitochondrial OXPHOS. We assume that liver mitochondrial OXPHOS directs much more ATP to gluconeogenesis than anabolic pathways like glycogen, triglyceride and protein synthesis in insulin resistance or insulin deficiency compared with healthy condition. Our data suggest induced liver OXPHOS, as a characteristic of diabetes, in return enhances hepatic gluconeogenesis and deteriorates the hyperglycaemia, becoming a pivotal process in the vicious circle of hyperglycaemia and gluconeogenesis (Figure 8B).

Our study also revealed that the changing pattern of liver OXPHOS function was quite different between type 1 and type 2 diabetes. The liver complex IV‐dependent respiration increased the most in type 1 diabetic mice. However, in type 2 diabetes, the alteration of liver OXPHOS in complex I was the most significant. Furthermore, OXPHOS function in liver steadily inclined during type 1 diabetes. In contrast, enhanced OXPHOS reached the peak at the onset of type 2 diabetes then declined to nearly normal level in the late stage of the disease (Figure 8A, which is based on the ratio of liver mitochondrial state 3 respiration of disease models to corresponding controls shown as Table S1). The different pattern of liver OXPHOS between type 1 and type 2 diabetes also reflects two distinct mechanisms. Burst of hyperglycaemia resulted from islet destruction fuels liver respiratory chain to work overload after type 1 diabetes onset. Parabola‐shaped alteration of liver OXPHOS in type 2 diabetes indicates the respiratory chain becomes overburdened long before disease onset. This study revealed the difference in liver mitochondrial function between type 1 and type 2 diabetes, and we demonstrated liver mitochondrial complex IV may be involved in the progression of type 1 diabetes. However, in most of published studies the complex IV‐dependent respiration was rarely investigated in metabolic disorders, so the clinical implication of this finding needs further exploration.

According to our in vitro data, only the inhibition of mitochondrial complex I rather than other complexes in the primary hepatocytes under normal and lipid accumulated condition markedly decreased glucose production without cytotoxicity, suggesting inhibitors of complex II, III, IV or V may not be safe or feasible for diabetes treatment. Our previous studies demonstrated that berberine, metformin and rotenone were able to lower glucose through inhibition of mitochondrial complex I.^44^, ^45^ Coincidentally, this study showed that the liver mitochondrial complex I‐dependent respiration was up‐regulated greatly in the pre‐diabetes and early stage of type 2 diabetes. Our findings provide pathophysiological evidence for the usage of complex I inhibitors as anti‐hyperglycaemic agents. In addition, our results warn that it should be prudent to prescribe these agents to aged patients with long duration of type 2 diabetes, because the induced liver mitochondrial complex I‐dependent respiration returned to nearly normal level as the disease progressed to the late stage.

Last but not least, the limitations of our study are as follows: (a) C57BL/6J mice instead of wt/db or wt/wt littermates were used as controls of db/db mice, because the littermates were not commercially available then; (b) the adenine nucleotide levels were only measured in early‐stage type 1 diabetic mice under fasted and anaesthetic condition, which might have an impact on the ratio of ATP/ADP; and (c) our experiment design included measuring state I, II, III respiration, OXPHOS capacity and ETC capacity, but mitochondrial respiratory control ratio (RCR), an important indicator of coupling, was not measured. We will include the RCR in our future study.

## 5 CONCLUSIONS

Our results demonstrated that the liver mitochondria were more sensitive and adaptive during metabolic disorders than skeletal muscle mitochondria. Over‐active complex IV and I of liver might be an important feature during the development of type 1 and type 2 diabetes, respectively. Moreover, our in vitro results indicated that inhibition of liver complex I rather than other complexes was able to ameliorate hyperglycaemia through suppressing hepatic glucose production through reducing cellular energy state.

## CONFLICT OF INTEREST

The authors have no conflicts of interest associated with this manuscript.

## AUTHORS’ CONTRIBUTIONS

JY is the guarantor of this work and, as such, had full access to all the data in the study and takes responsibility for the integrity of the data and the accuracy of the data analysis. JY designed the studies. MA carried out the research. XY, MY and WH assisted in performing research. ZY performed the determining the content of adenine nucleotides by HPLC. MA and JY interpreted the results and wrote the manuscript. YB and YY assisted in reviewing and revising the manuscript. All authors approved the final version of the manuscript.

## Supporting information

Supplementary Material

## Data Availability

All data generated or analysed during this study are included in this published article.
