# Supplementary material for: Enhanced liver but not muscle OXPHOS in diabetes and reduced glucose output by complex I inhibition
Source: J Cell Mol Med. 2020 Apr 6;24(10):5758–71. doi: 10.1111/jcmm.15238 (PMC7214161; doi:10.1111/jcmm.15238)
Supplement: Supplementary file 1 — Supplementary Material [file JCMM-24-5758-s001.docx]

**Supplementary Data**

**Supplemental Table 1**. **The fold changes of liver mitochondrial OXPHOS function of the disease models relative to the control.**

|  |  | Fold change of OXPHOS function to control | | | | |
| --- | --- | --- | --- | --- | --- | --- |
|  | Respiratory state | Early stage of type 1  diabetes | End stage of type 1 diabetes | Pre-diabetes (HFD) | Early stage of type 2 diabetes | End stage of type 2 diabetes |
| Complex I dependent | State 2 | 1.26±0.30 | 1.33±0.15 | 1.43±0.14 | 1.75±0.13^**^ | 0.91±0.10 |
|  | State 3 | 1.26±0.19^*^ | 1.37±0.13 | 1.76±0.16^*^ | 1.48±0.07^**^ | 0.90±0.09 |
|  | OXPHOS Capacity | 1.17±0.21^*^ | 1.34±0.11 | 1.98±0.21^**^ | 1.81±0.23^**^ | 1.03±0.09 |
|  | ETC Capacity | 1.17±0.24^*^ | 1.20±0.15 | 1.88±0.17^**^ | 1.72±0.20^**^ | 1.06±0.11 |
| Complex II dependent | State 2 | 1.14±0.08 | 1.25±0.14 | 0.73±0.08 | 1.26±0.11 | 1.77±0.27 |
|  | State 3 | 1.38±0.12^*^ | 1.54±0.23 | 1.29±0.13 | 1.41±0.10^**^ | 1.22±0.09 |
|  | OXPHOS Capacity | 1.38±0.07^*^ | 1.46±0.23 | 1.46±0.22 | 1.56±0.17^*^ | 1.25±0.1 |
|  | ETC Capacity | 1.42±0.08^*^ | 1.48±0.26 | 1.55±0.12^**^ | 1.48±0.15^*^ | 1.41±0.13^*^ |
| Complex Ⅳ dependent | TMPD/Asc | 1.46±0.06^***^ | 1.72±0.20^**^ | 1.21±0.10 | 1.10±0.11 | 1.06±0.04 |
|  | OXPHOS Capacity | 1.50±0.11^**^ | 2.22±0.32^**^ | 0.83±0.11 | 1.01±0.09 | 0.88±0.11 |

Data are expressed as means ± SEM (*n* = 4-15). ** P< 0.05, **P < 0.01, ***P < 0.001* *vs.* corresponding control.


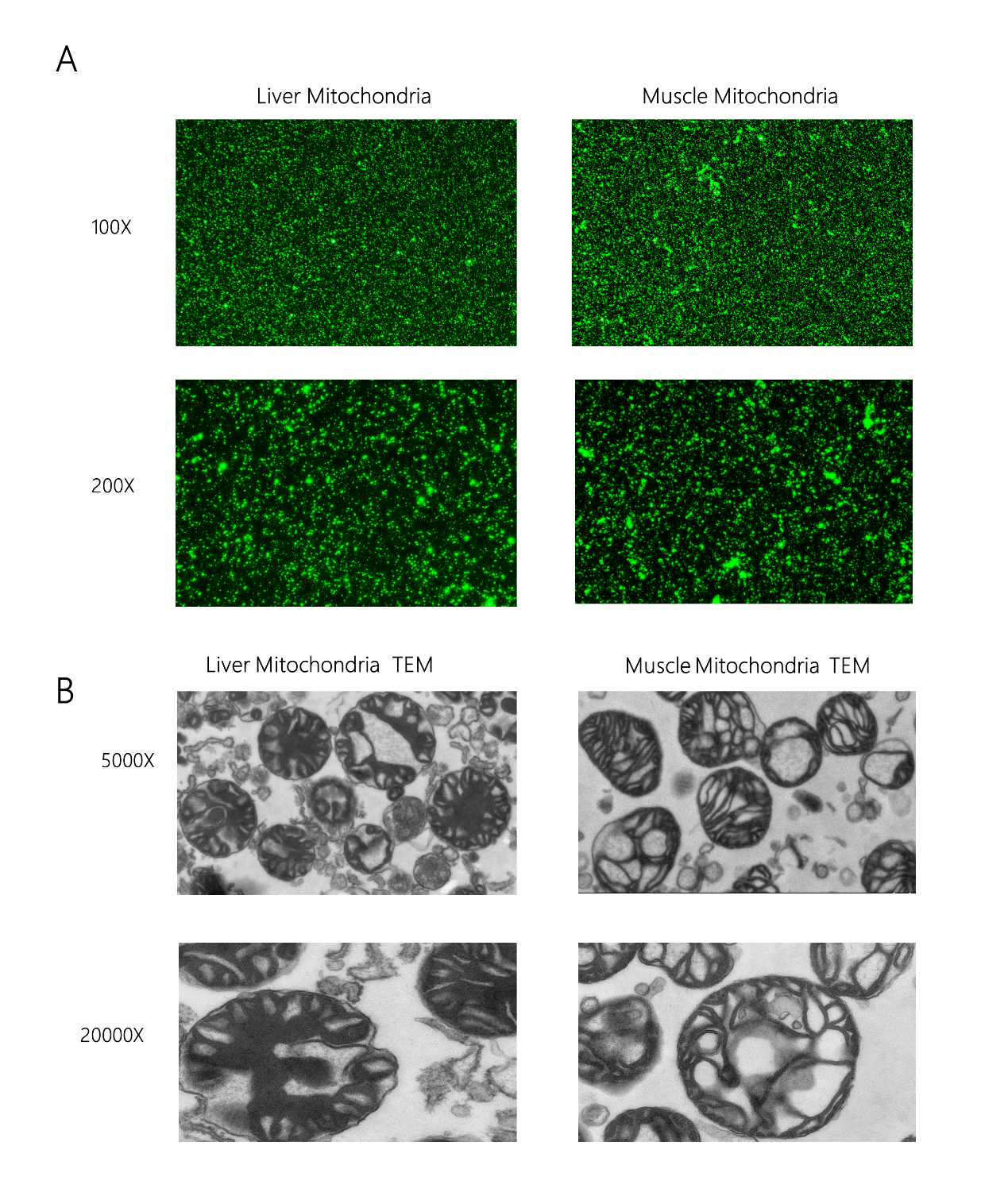


**Supplemental Figure 1.** A: The representative image of isolated liver (left) and muscle (right) mitochondria stained with 0.5 μmol/L Mitotracker Green. B: The representative TEM image of isolated liver (left) and muscle (right) mitochondria.


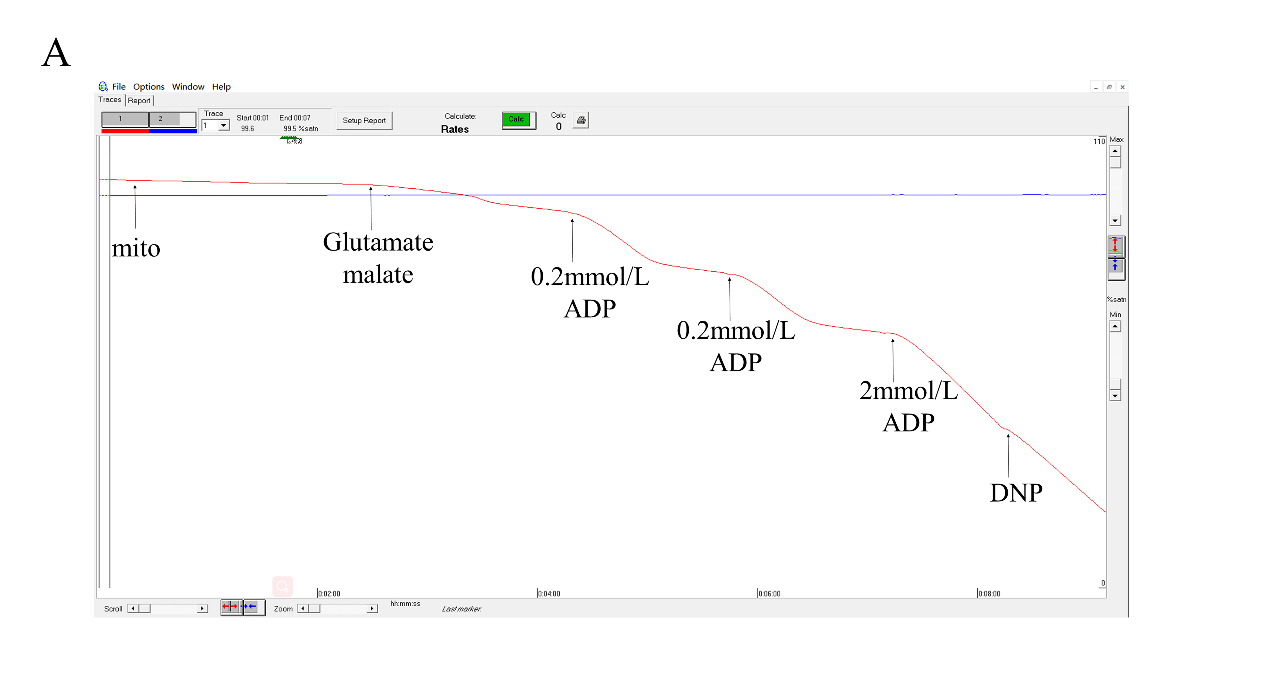


**Supplemental Figure 2.** A representative trace of recording of complex I-dependent OCR.

**
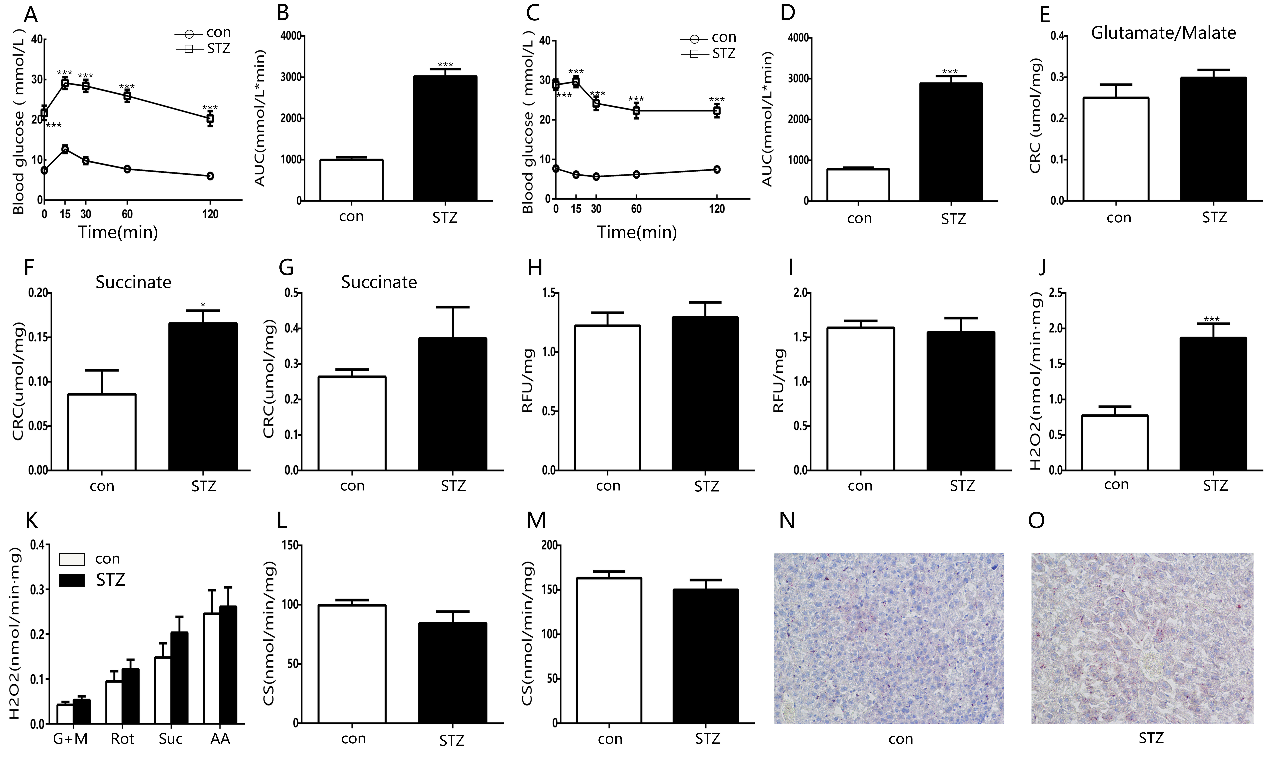
**

**Supplemental Figure 3.** Supplemental data of early stage type 1 diabetic and control mice. The IPGTT for early stage type 1 diabetic and control mice. B: The area under curve (AUC) of IPGTT. C: The ITT for early stage type 1 diabetic and control mice. D: The AUC of ITT. E-G: The complex I-dependent calcium CRC of liver mitochondria (E), and complex II-dependent CRC of liver (F) and muscle (G) mitochondria. H-I: The liver mitochondrial complex I-dependent membrane potential (H) and muscle mitochondrial complex I-dependent membrane potential (I). J-K: The liver mitochondrial H_2_O_2_ production (J) and muscle mitochondrial H_2_O_2_ production (K). L-M: The CS activity of liver (L) and muscle (M) mitochondria isolated from early stage type 1 diabetic and control mice. N-O: Representative images show oil red O staining of liver sections from control (N) and early stage STZ-induced type 1 diabetic (O) mice. Data are expressed as means ± SEM (*n* = 8). * *P*< 0.05*,* ***P* < 0.01, ****P* < 0.001 *vs.* control.


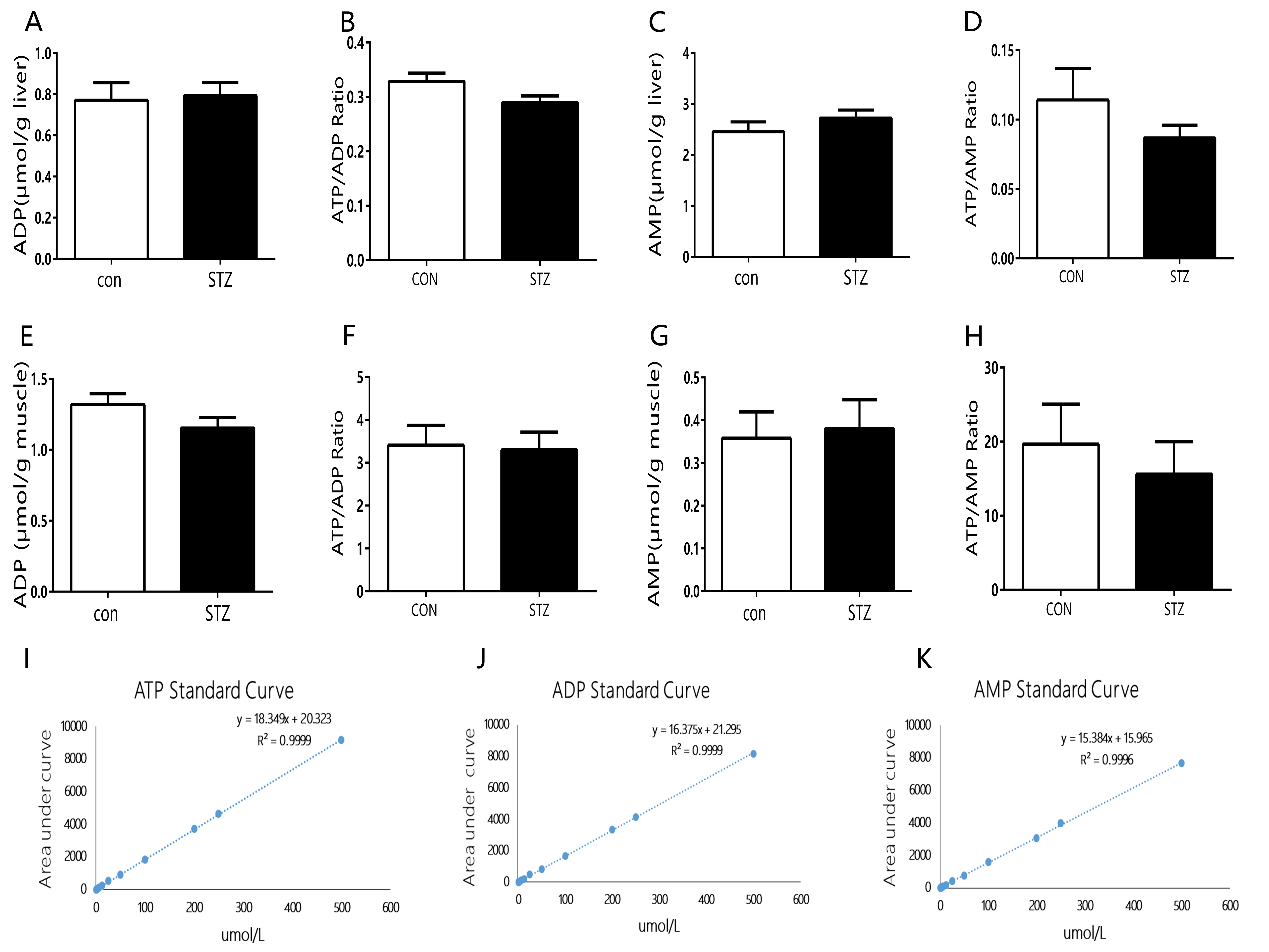


**Supplemental Figure 4.** The adenosine phosphate content of liver and muscle did not change in early stage STZ-induced type 1 diabetic mice. A and C: The content of ADP and AMP of liver from early stage type 1 diabetic and their corresponding control mice. B and D: The ATP/ADP and ATP/AMP ratio of liver. E and G: The content of ADP and AMP of muscle from early stage type 1 diabetic and their corresponding control mice. F and H: The ATP/ADP and ATP/AMP ratio of muscle. I: The standard curve of ATP; J: The standard curve of ADP; K: The standard curve of AMP.


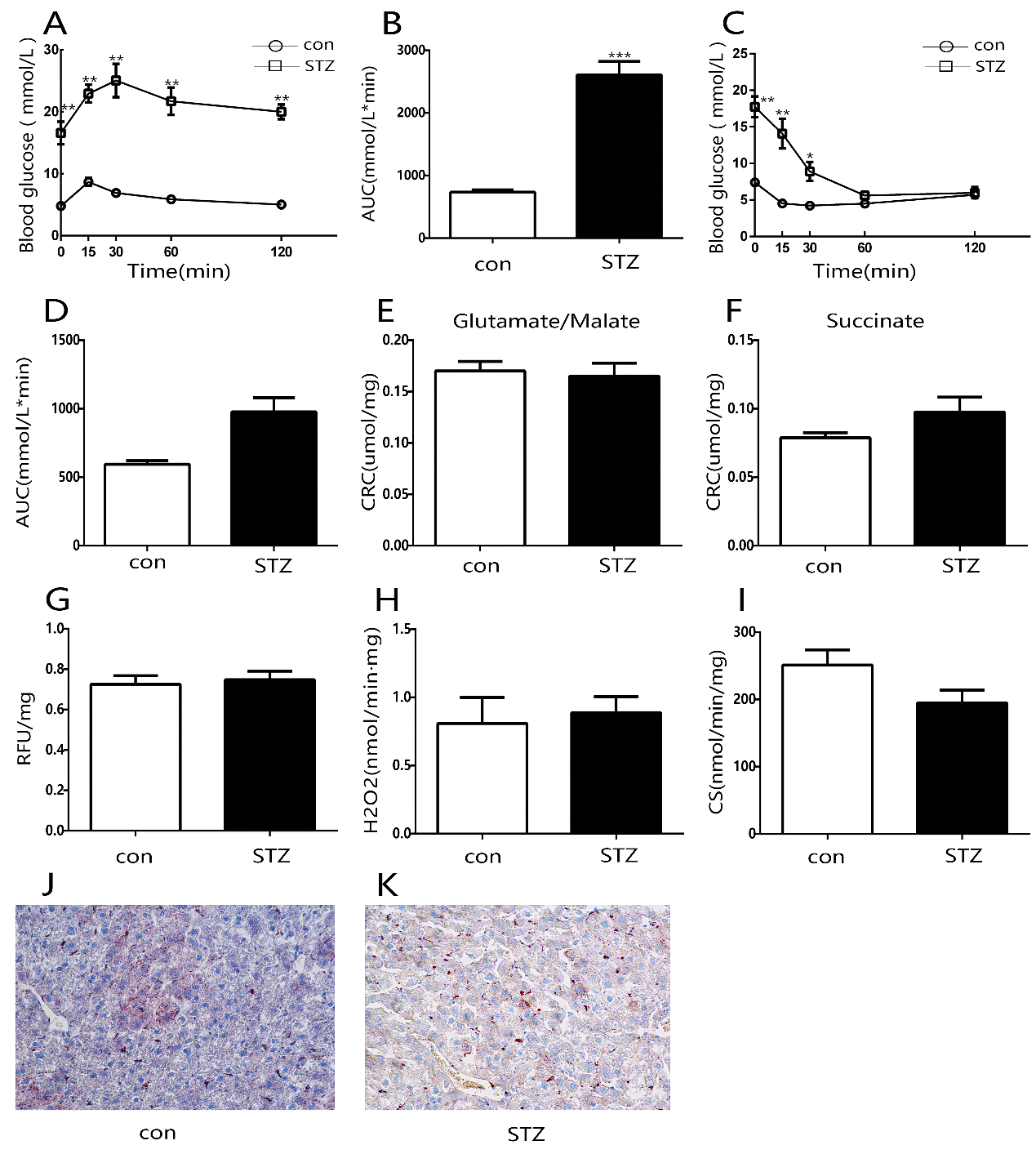


**Supplemental Figure 5.** Supplemental data of late stage STZ-induced type 1 diabetic and control mice. A: The IPGTT for late stage type 1 diabetic and control mice. B: The AUC of IPGTT. C: The ITT for late stage type 1 diabetic and control mice. D: The AUC of ITT. E: The complex I-dependent CRC of liver mitochondria. F: The complex II-dependent CRC of liver mitochondria. G: The liver mitochondrial complex I-dependent membrane potential; H: The liver mitochondrial H_2_O_2_ production. I: The CS activity of liver mitochondria isolated from late stage type 1 diabetic and control mice. Data are expressed as means ± SEM (*n* = 5). * *P*< 0.05*, vs.* control.


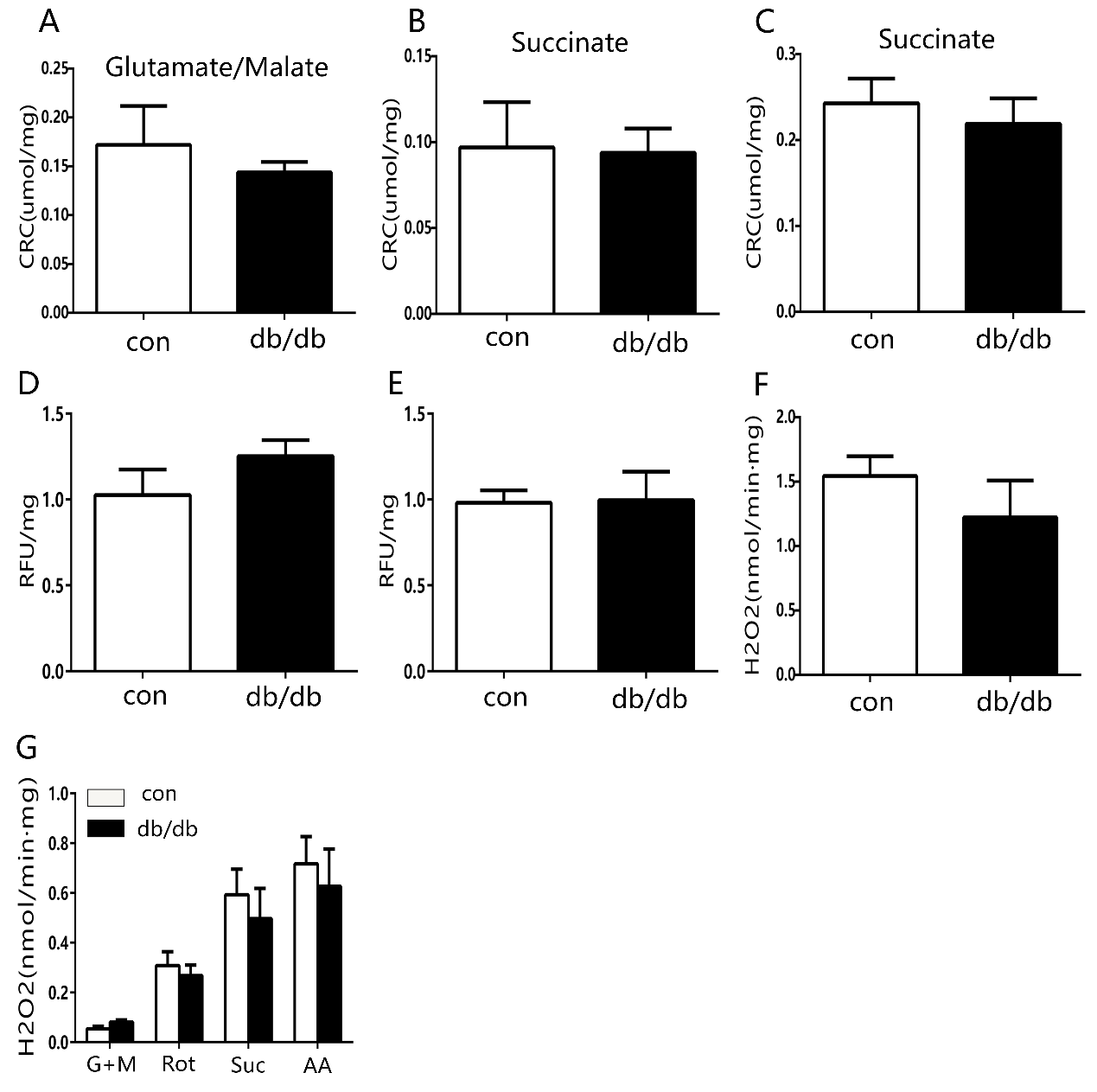


**Supplemental Figure 6.** Supplemental data of early stage type 2 diabetic and control mice. A-C: The complex I-dependent CRC of liver mitochondria (A), and complex II-dependent CRC of liver (B) and muscle (C) mitochondria. D-E: The liver mitochondrial complex I-dependent membrane potential (D) and muscle mitochondrial complex I-dependent membrane potential (E). F-G: The liver mitochondrial H_2_O_2_ production (F) and muscle mitochondrial H_2_O_2_ production (G). Data are expressed as means ± SEM (*n* = 8). * *P*< 0.05, ***P <* 0.01, ****P* < 0.001 *vs.* control.


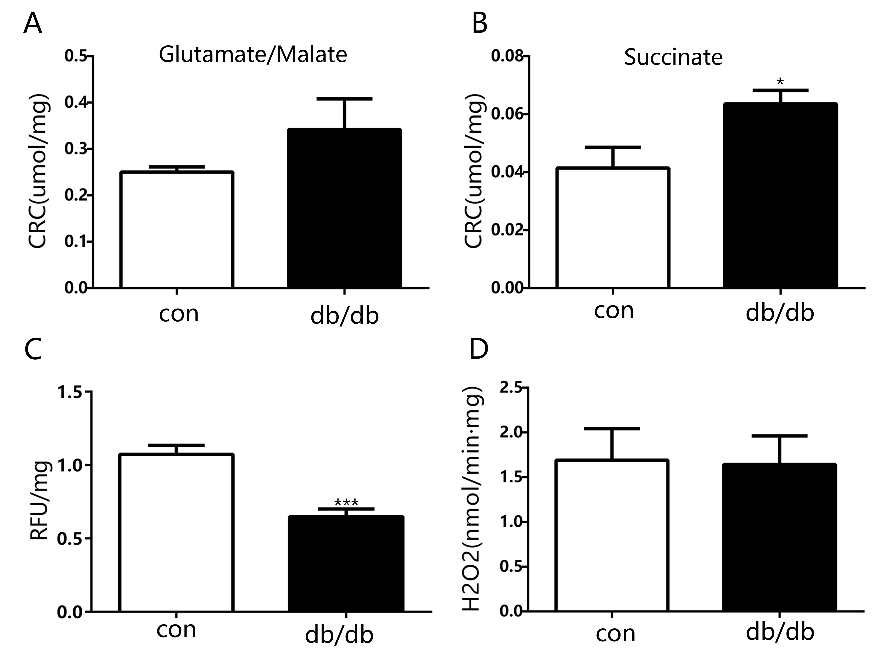


**Supplemental Figure 7.** Supplemental data of late stage type 2 diabetic db/db and control mice. A: The complex I-dependent CRC of liver mitochondria; B: The complex II-dependent CRC of liver mitochondria; C: The liver mitochondrial complex I-dependent membrane potential; D: The liver mitochondrial H_2_O_2_ production. Data are expressed as means ± SEM (*n* = 4-8). * *P*< 0.05, *vs.* control.

**
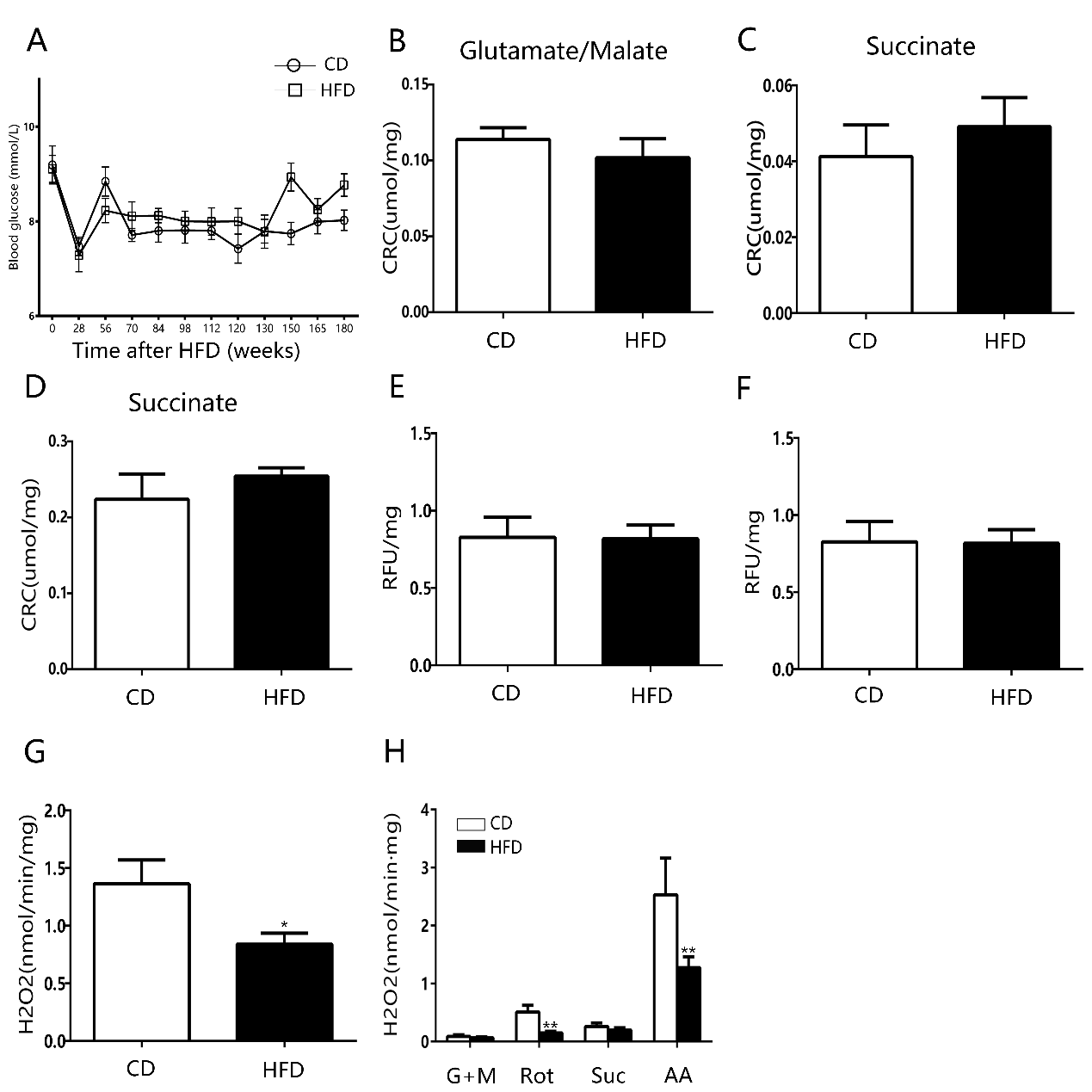
**

**Supplemental Figure 8.** Supplemental data of HFD and CD fed mice. A: The random blood glucose of HFD and CD mice. B-D: The complex I-dependent CRC of liver mitochondria (B), and complex-II dependent CRC of liver (C) and muscle (D) mitochondria. E-F: The liver mitochondrial complex I-dependent membrane potential (E) and muscle mitochondrial complex I-dependent membrane potential (F). G-H: The liver mitochondrial H_2_O_2_ production (G) and muscle mitochondrial H_2_O_2_ production (H). Data were expressed as means ± SEM (*n* = 10-15). * *P*< 0.05, ***P* < 0.01, ****P* < 0.001 *vs.* control.


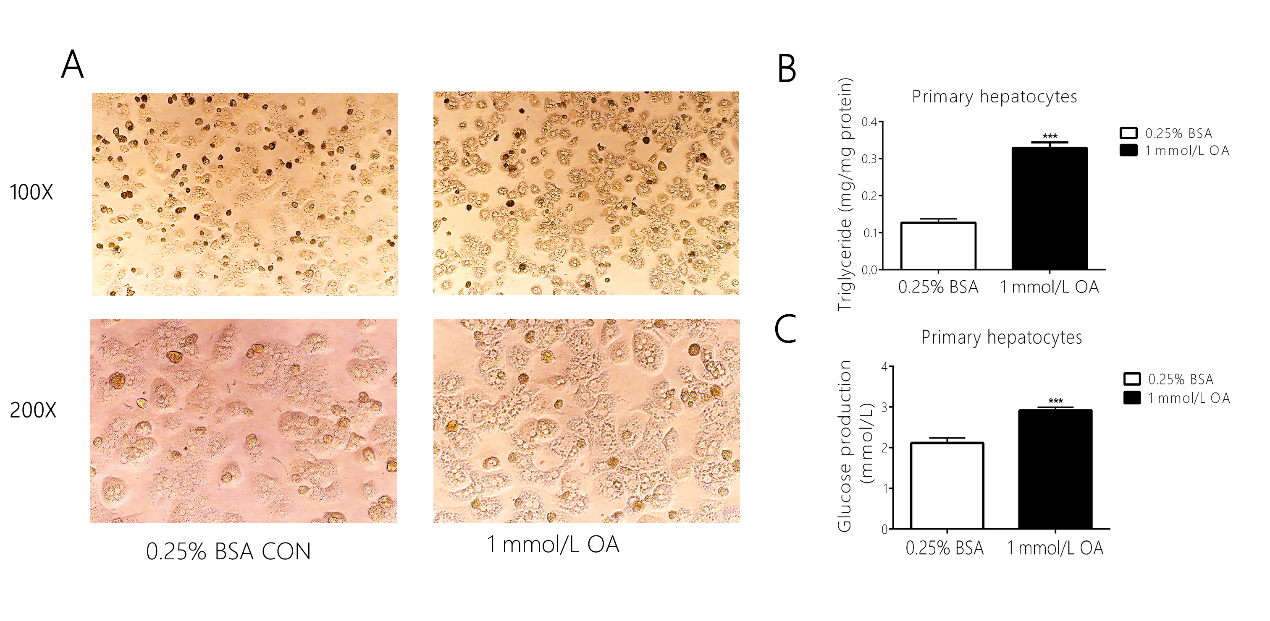


**Supplemental Figure** 9. Establishment of the primary hepatocytes with lipid accumulation. A: The representative light microscope images of primary hepatocytes treated with either 0.25% BSA or 1 mmol/L OA for 20h; B: The triacylglyceride content of primary hepatocytes treated with 0.25% BSA or 1 mmol/L OA for 20h; C: The glucose production of primary hepatocytes pre-treated with 0.25% BSA or 1 mmol/L OA for 20h.

**
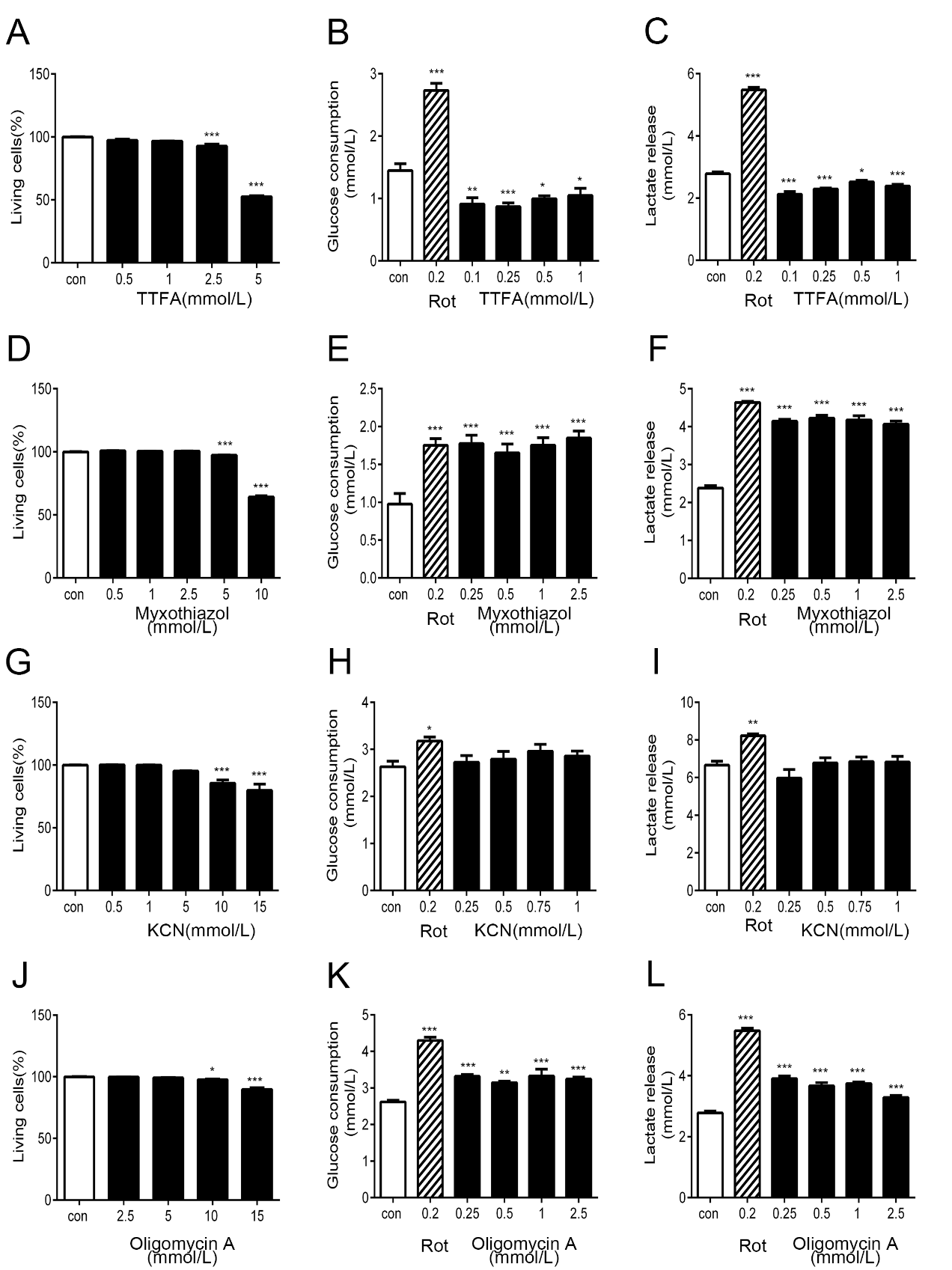
**

**Supplemental Figure 10.** The effect of each complex inhibitor on glycolysis and cytotoxicity in HepG2 cells. The living cell percentage (A), glucose consumption (B) and lactate release (C) of HepG2 cells after treated with different concentration of TTFA. The living cell percentage (D), glucose consumption (E) and lactate release (F) of HepG2 cells after treated with different concentration of myxothiazol. The living cell percentage (G), glucose consumption (H) and lactate release (I) of HepG2 cells after treated with different concentration of KCN. The living cell percentage (J), glucose consumption (K) and lactate release (L) of HepG2 cells after treated with different concentration of oligomycin A. Data are expressed as means ± SEM (*n* = 8). ^*^ *P*< 0*.*05, ^**^*P* < 0*.*01, ^***^*P* < 0*.*001 *vs.* control.


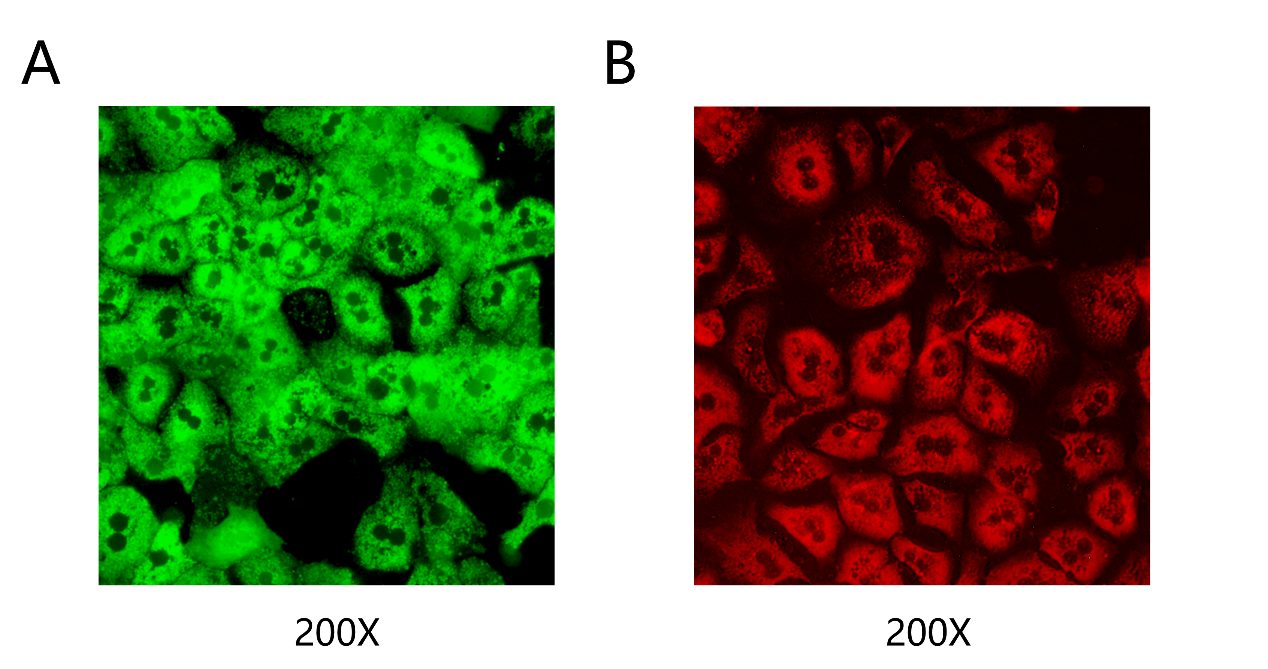


**Supplemental Figure 11.** A: The representative image of mouse primary hepatocytes treated with 20 μmol/L DCFDA for 45 min after incubation in glucose output medium for 6h. B: The representative image of mouse primary hepatocytes treated with 2.5 μmol/L Mitosox Red for 10 min after incubation in glucose output medium for 6h.
